# The genomes sequenced for the neotropical stingless bees *Scaptotrigona bipunctata* and *S. depilis* strengthen the phylogenomics support for the taxonomy of social bees

**DOI:** 10.1590/1678-4685-GMB-2024-0255

**Published:** 2025-11-28

**Authors:** Paulo Gonzalez Hofstatter, Flávia Cristina de Paula Freitas, Danielle Luna-Lucena, Leonardo Campana, Klaus Hartfelder

**Affiliations:** 1 Universidade de São Paulo, Faculdade de Medicina de Ribeirão Preto, Departamento de Biologia Molecular e Celular e Bioagentes Patogênicos, Ribeirão Preto, SP, Brazil.; 2 Universidade Federal de São Carlos, Centro de Ciências Biológicas e da Saúde, São Carlos, SP, Brazil.; 3 Universidade Estadual de Campinas, Instituto de Biologia, Departamento de Bioquímica e Biologia Tecidual, Campinas, SP, Brazil.; 4 Universidade de São Paulo, Faculdade de Medicina de Ribeirão Preto, Departamento de Genética, Ribeirão Preto, SP, Brazil.

**Keywords:** Social bees, genome assembly, Meliponini, Scaptotrigona, corbiculate bees

## Abstract

Bees are fundamental factors in ecology and agriculture due to their ecosystem services as pollinators, including many important crops. Because of its ecological significance and value to humans, the honey bee, *Apis mellifera*, was one of the earliest insect species targeted for genome sequencing, and over the last decades, many other species of social bees, including practically all species comprising the genus *Apis* and dozens of bumble bee species (Bombini) have complete genome assemblies deposited in public databases. The largest clade of the social bees, the stingless bees (Meliponini), is, however, strongly underrepresented. To date, only five genomes for species of three genera have been released for the New World stingless bees, which comprise over 400 species distributed in 32 genera. Different from the honey bee, these species are native to the Neotropics, being important pollinators of many native plants and cultivars, including greenhouse cultures. We present here the genome assemblies for two species of the genus *Scaptotrigona*, one of the largest genera among the stingless bees in Brazil. The new datasets are highly complete and, as shown in our phylogenomics analysis, these genomes provide robust support for the clades of the corbiculate bees and their evolutionary history.

## Introduction

Pollination provided by bees is a key ecosystem service for both wild flowers and many crops. For the latter, bees generate revenues in the billion-dollar range worldwide (Klein et al., 2007; Gallai et al., 2009; Baylis et al., 2021). For Brazil, Giannini et al. (2015) estimated at US$ 12 billion per year the impact of pollinators for 85 crops. For South America, a recent estimate calculates this as a US$ 22.95 billion annual contribution (Basualdo et al., 2022). Bees are the key providers of this ecosystem service, yet their populations are reported to undergo declines worldwide, associated with changes in land use and agricultural practice (Murray et al., 2009; Odanaka and Rehan, 2019; Janousek et al., 2023; Richardson et al., 2023).

Among the social bees, the Western honey bee, *Apis mellifera*, is clearly the most intensively studied species. To the New World, however, the honey bee is a newcomer, having been introduced both to North and South America primarily during the last two centuries. The main group of social bees in the Americas, the stingless bees (Meliponini), has a long tradition in beekeeping by indigenous people (Vit, 2013; Quezada-Euán et al., 2018). Both the honey bees and the stingless bees live in colonies with a high eusocial organization. As such, they have a morphologically defined female caste system, where each colony is composed of hundreds to thousands of subfertile workers that perform all the colony maintenance tasks, and a single, highly fertile queen. Males are only temporarily present in the colonies, as already within a few days after emergence from their brood cells, they leave to mate with a young, virgin queen from another colony (Vollet-Neto et al., 2018). 

The Neotropical solitary to facultatively social orchid bees (Euglossini), the primitively eusocial bumble bees (Bombini) prevalent in temperate climates, the Old-World honey bees (Apini), and the pantropical stingless bees (Meliponini) together form the clade of corbiculate bees. The honey bees, composed of the single genus *Apis* with only 10-12 recognized species (Engel, 1999; Lo et al., 2010), and the stingless bees are the only two clades that reached the peak of social organization. But different from the honey bees, the tribe Meliponini is highly diverse and distributed worldwide. Estimates vary from around 550 species in 58 genera (Grüter, 2020) to over 600 species distributed in 45 genera (Engel *et al.*, 2023). The largest number of the genera (32) and taxonomically recognized species (417) (Camargo and Pedro, 2007) occur in the Neotropics (Michener, 2007), but ongoing population genetics studies indicate that the number of species is likely even higher. A detailed key to the genera and subgenera of the Neotropical Meliponini has recently been provided by Engel *et al.* (2023), and the phylogenomics study by Lepeco et al. (2024) based on the sequencing of ultraconserved genome elements provides strong support to the earlier published evolutionary history of the stingless bees (Rasmussen and Cameron, 2010). 

The currently most accepted phylogeny of the corbiculate bees places the Meliponini as sister group to the Bombini, with Apini as sister clade to both (Bossert et al., 2019; Almeida et al., 2023; Lepeco et al., 2024). Implicitly, this would be indicative of an independent origin of the highly advanced eusocial organization for the honey bees and stingless bees. Rasmussen and Cameron (2010) inferred the origin of the stingless bees and the subsequent split between the Neotropical and the Indio-Malayan/Australian/African genera to have occurred in the Upper Cretaceous (66-95 million years ago, Mya). The subsequent radiation of the Neotropical stingless bees as an isolated group was estimated by the same authors to have started soon after this split. In comparison, the honey bees (Apini) have a much more recent history of diversification, during the last 22 Mya (Cardinal et al., 2010). 

Though much more species-rich, biologically highly diverse, and also more ancient than the honey bees, this diversity is not reflected in the knowledge about their genomes. The genome of the Western honey bee, *Apis mellifera*, was actually the third insect genome to be fully sequenced (The Honey Bee Genome Consortium, 2006), and practically all genomes of the order Hymenoptera sequenced thereafter used the honey bee genome as reference. Currently, the genomes for six (of the 10) *Apis* species are completely sequenced and available in GenBank, and so are 35 genomes of the approximately 250 species of the tribe Bombini. However, for the primarily Neotropical orchid bees (Euglossini), genomic information is available for only two species of the over 250 species, and for the Meliponini, only 12 of the over 550 species have genome sequences deposited, and only five of these are Neotropical species. 

The first of the five published Neotropical Meliponini genomes was that of *Melipona quadrifasciata*, popularly known in Brazil as “mandaçaia”, which was included in the comparative genomics study of Kapheim et al. (2015) that aimed to identify genomic hallmarks in the evolution of the bees from an ancestral solitary life to the highly advanced eusociality. *M. quadrifasciata* is an emblematic species, because its caste system is considered to be genetically determined (Kerr, 1950). The next sequenced stingless bee genome was that of *Frieseomelitta varia* (de Paula Freitas et al., 2020), known as “marmelada”. This species was of interest because, different from most of the other stingless bees, its workers are considered to be completely sterile. In 2024, two new stingless bee genome assemblies were reported. First, the chromosome-level assembly of the *Melipona bicolor* genome (Araujo *et al.*, 2024), which is of biological interest because it is one of the few species for which the coexistence of two or more queens in a colony (polygyny) is known (Velthuis et al., 2006), and the second was the genome dataset for *Tetragonisca angustula* (Ferrari et al., 2024). Popularly known as ‘jataí’, this species has a wide distribution in Brazil and is highly valued for the medicinal properties of its honey. Another genome of a Neotropical stingless bee, that of *Melipona beechei*, has been deposited in GenBank and is mentioned in a study on venom protein genes present in stingless bees (Koludarov et al., 2023), together with the genome of the Australasian species *Tetragonula carbonaria*. Unpublished genomes of six other Australasian Meliponini deposited in GenBank, generally from short reads assemblies, were recently comparatively analyzed for completeness (Araujo *et al.*, 2024). 

Here, we now report scaffold-level genome assemblies based on PacBio long reads for two species of the genus *Scaptotrigona*. These species were selected for sequencing because *Scaptotrigona* is one of the largest genera of the Neotropical stingless bees, alongside *Melipona*, *Plebeia* and *Trigona*, and because they are gaining increasing interest in Brazil as pollinators, particularly for high-value greenhouse crops (Bomfim et al., 2014; Osterman et al., 2021; Ramos et al., 2024). 

## Material and Methods

### Collection of haploid males

**Figure 1 -:**
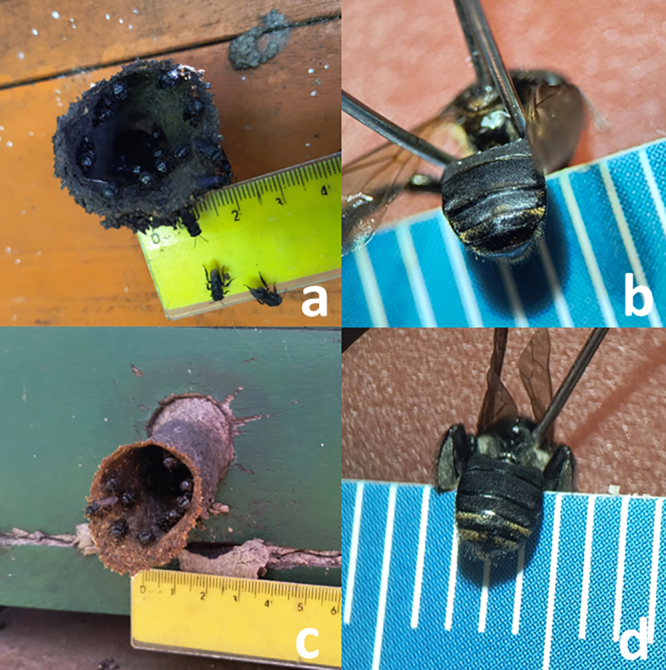
Species used for this project: a, b) *Scaptotrigona bipunctata*: nest entrance with guard bees (a), and taxonomically relevant characteristics on abdomen (b). c, d) *Scaptotrigona depilis*: nest entrance with guard bees (c), and taxonomically relevant characteristics on abdomen (d).

Specimens were collected from a single colony each of *Scaptotrigona bipunctata* Lepeletier, 1836 and *Scaptotrigona depilis* Moure, 1942. The colonies are kept in experimental hives in the bee yard of the campus of the University of São Paulo in Ribeirão Preto, SP, Brazil (Figure 1). The colonies are of local origin and were originally obtained from their natural nesting sites in trees of the campus, and were eventually transferred to the bee yard when the trees had to be cut. For this study we collected brown-eyed male pupae, which are the haploid sex. We preferred the sampling of pupae, as this avoided microbial contamination in the genome assemblies, because their guts had just been newly formed during metamorphosis and, hence, were still microbiota free. Immediately after the collection, the individuals were snap-frozen in liquid nitrogen and stored at -80 °C for further processing. Voucher specimens consisting of adult workers collected simultaneously from these colonies were deposited in the Coleção Entomológica Prof. J.M.F. Camargo of the Faculdade de Filosofia, Ciências e Letras de Ribeirão Preto - Universidade de Ribeirão Preto (*S*. *bipunctata* voucher USP_RPSP 00013262; *S. depilis*USP_RPSP 00014020). The voucher specimens were identified by the curator of the collection, Prof. Dr. Eduardo A.B. de Almeida. The maintenance of the bee colonies in the meliponary of the Ribeirão Preto Campus of the University of São Paulo is authorized by the *Instituto Chico Mendes de Conservação de Biodiversidade - Ministério de Meio Ambiente*, Brazil, under the license 81843-1.

### DNA preparation and sequencing 

High molecular weight DNA was extracted using the MagAttract HMW DNA kit (Qiagen, Hilden, Germany) following the manufacturer’s protocol. Briefly, repeated samples consisting of two males each were transferred to 2 mL Eppendorf tubes containing lysis buffer provided by the DNA extraction kit and incubated in a thermoMixer (Eppendorf, Hamburg, Germany) at 56 °C for 16 h. Insoluble material was removed by centrifugation at 10,000 × *g* for 5 min at RT. RNase, extraction buffers, and MagAttract Suspension magnetic particles, all provided by the kit, were added to the supernatant, and the samples were incubated in a thermomixer at RT for 3 min at 1400 rpm. Placed in the holder of a magnetic rack (GE Health Care, Chicago, IL, USA), the supernatant was removed from the samples, and two washing cycles with wash buffer were performed, each consisting of an incubation in the thermomixer (3 min at 1400 rpm each) followed by magnetic separation. Subsequently, the magnetic particles were carefully rinsed with distilled water while still on the magnetic rack, and after removal of all the water, 100 µl of elution buffer were added to the magnetic particles. After incubation for 3 min at 1400 rpm the vials were again placed on the magnetic rack, the eluted DNA was removed to a new 1.5 mL Eppendorf vial, and the elution step was repeated by adding another 100 µl of elution buffer to the magnetic particles. The quality and quantity of the extracted DNA was assessed by spectrophotometry readings at 260/280 nm (Nanoview, GE Health Care) and in a Qubit 4.0 reader using the Standard DNA broad range mix (Thermo-Fisher, Waltham CA, USA). For sequencing, which required a total of 10 µg of DNA, three samples of each extraction procedure were pooled per species.

Library construction and PacBio sequencing were performed by the SENAI/CETIQT facility, Rio de Janeiro, Brazil, where prior to sequencing the HMW DNA quality was checked by pulse-field electrophoresis (Pippin Pulse system). After SMRTbell library preparation, High Fidelity long read sequencing was done using a SMRT Cell 8M (PacBio, Menlo Park, CA, USA) on a PacBio Sequel II instrument. 

### Genome size estimation, assembly, and synteny analysis

The sizes of the two genomes were estimated bioinformatically using FindGSE (Sun et al. 2018). We used three different assemblers for this process: the Hicanu tool of the Canu assembler program (Koren et al., 2017; Nurk et al. 2020), Hifiasm (Cheng et al. 2021), and Flye (Kolmogorov et al., 2019). As the assemblers Hifiasm and Flye produced better results in terms of contiguity, they were the ones used in the downstream analyses, where they were merged with the software *quickmerge* (Chakraborty et al., 2016) to further improve contiguity. Quast (Gurevich et al., 2013) was employed to evaluate genome quality and contiguity. These first assembly results yielded scaffolds of Mb size. However, because of the heterozygosity in the sample, due to the need for pooling the DNA from several haploid males, the assembly presented a mixture of different haplotypes that had to be subjected to a purging step. For this we employed the protocol *purge-haplotigs* (Roach et al. 2018) to obtain haploid level datasets. After purging, the datasets presented low levels of duplication, as assessed by BUSCO (Seppey et al. 2019). We also used BUSCO for assessing the completeness of the assemblies (using Hymenoptera as a reference lineage). Final polishing of the assemblies was performed with Pilon (Walker et al., 2014). CG content was estimated with the Bbmap tool *stats.sh* (Buschnell, 2014), and synteny analyses were performed using the online tool D-Genies (Cabanettes and Klopp, 2018).

### Gene prediction

We used two complementary approaches for gene prediction, first *ab initio* and then one based on RNA-seq evidence. First, we used RepeatModeler (Flynn et al., 2020) and RepeatMasker (Smit *et al.* 2015) for masking repetitive regions of the genome. RepeatModeler identifies different families of repetitive elements with the Dfam/Repbase databases, whereas RepeatMasker masks the genome with the fasta custom file of the identified families. For the *ab initio* gene prediction, we then used the Augustus program (Stanke et al. 2006) with *Apis mellifera* as reference. This yielded a GFF3 annotation file for the protein coding genes (CDS) and their respective proteins for each species. The following step was then used to run BUSCO again for assessing completeness of the resulting files.

In the second gene prediction approach, we incorporated RNA-seq data from ovaries, brains and fat body of *Scaptotrigona bipunctata* workers, generated by ongoing projects in our laboratory. The RNA-seq data was mapped to the *S. bipunctata* genome using STAR with default parameters (Dobin et al. 2013) and used as input to the BRAKER2 pipeline (Stanke et al., 2006, 2008; Bruna et al., 2021), which produced a more complete gene prediction now based on RNA-seq evidence. However, due to the presence of multiple RNA isoforms, the gene prediction process resulted in redundancy for some genes, which could interfere with the downstream phylogenomics analysis. To address this, we used the results of the first gene prediction approach in the phylogenomics analysis to avoid redundancy generated by the presence of mRNA isoforms in the second approach. To reduce redundancy in other datasets obtained from public databases and our own, we employed CD-HIT clustering (Fu et al. 2012).

### Phylogenomic analysis

A database composed of genomes, proteomes and transcriptomes comprising a diversity of bees was implemented locally with representatives of the four tribes of the corbiculate bees, namely Euglossini, Apini, Bombini and Meliponini, as well as solitary bee species not belonging to the corbiculate bees (Table S1). The datasets for the two *Scaptotrigona* species were produced in this study, whereas all the others were obtained from the publicly available NCBI repository, and annotated locally with Augustus (Stanke and Morgenstern, 2005) using *Apis mellifera* as reference. In the in-house pipeline for phylogenomics we used BUSCO to find single copy genes/proteins, the *esl-sfetch* tool from HMMER (Eddy 2011) to extract the sequences from datasets, *mafft* (Katoh and Standley 2013) to align the multifasta files, *trimal* (Capella-Gutiérrez et al. 2009) for trimming the non-aligned columns, and the perl program *catfasta2phyml.pl* (https://github.com/nylander/catfasta2phyml) for concatenating the trimmed MSA files into a single matrix. IQ-Tree2 (Minh et al. 2020) was used for phylogeny reconstruction, with 1000 bootstrap pseudoreplicates. IQ-Tree2 is a software based on the maximum likelihood approach and performed the tree reconstruction based on the substitution model GTR+F+I+G4, chosen automatically by the program.

## Results

### Genome assembly and preliminary dataset analyses

Hifi-PacBio sequencing generated 9.6 Gb of data for *Scaptotrigona bipunctata* and 8.3 Gb for *S. depilis*. Based on bioinformatics estimates, the genome sizes were approximately 325 Mb for *S. bipunctata* and 332 Mb for *S. depilis* (Figures S1 and S2), with the reads representing about ~30× coverage for the haploid genomes. Flye produced a dataset with low levels of duplication, while Hifiasm produced a dataset with a higher level of redundancy. Redundancy was removed through a purging process, reducing duplication from almost 10% to less than 0.4%, while completeness increased from 96% to over 98%. After the purging of haplotigs, merging and polishing with the original reads, we obtained a genome assembly of 301.7 Mb for *S. bipunctata* and 292.6 Mb for *S. depilis* (Table 1). The assembled genomes are deposited in GenBank under the Biosample accession numbers SAMN44369367, SAMN44369368 respectively.

**Table 1 -:** Comparison between the genome assemblies for the two *Scaptotrigona* species with the high-quality datasets for the model species *Apis mellifera* and *Bombus terrestris*, Chromosome numbers are based on Cunha et al. (2021).

Tribe	Meliponini		Apini	Bombini
Genus	*Scaptotrigona*		*Apis*	*Bombus*
Species	*S. bipunctata*	*S. depilis*	*A. mellifera*	*B. terrestris*
Haploid number	17	17	16	18
Assembly size	300.7 Mb	292.6 Mb	228 Mb	393 Mb
CG content	37.45%	37.52%	32.53%	38.7%
N50	11.3 Mb	12.9 Mb	13.6 Mb	14.6 Mb
Largest scaffold	21 Mb	19.5 Mb	27.7 Mb	25.5 Mb
Number of sequences	419	280	177	249
Coding genes	17,466	16,166	9,935	10,310
BUSCO completeness	98.4%	98.1%	98.4%	98.3%

The mitochondrial genomes for both species were also assembled and deposited in NCBI (accession numbers PV624662 for *S. bipunctata* and PV660477 for *S. depilis*). The assembled mitochondrial genome of *S. bipunctata* yielded a 14,938 bp sequence (Figure S3), while that of *S. depilis* resulted in a 15,130 bp sequence (Figure S4). These sizes are comparable to those observed in related bee species, such as *Melipona bicolor* (15 kbp) and *M. scutellaris* (14.8 kbp). 

**Figure 2 -:**
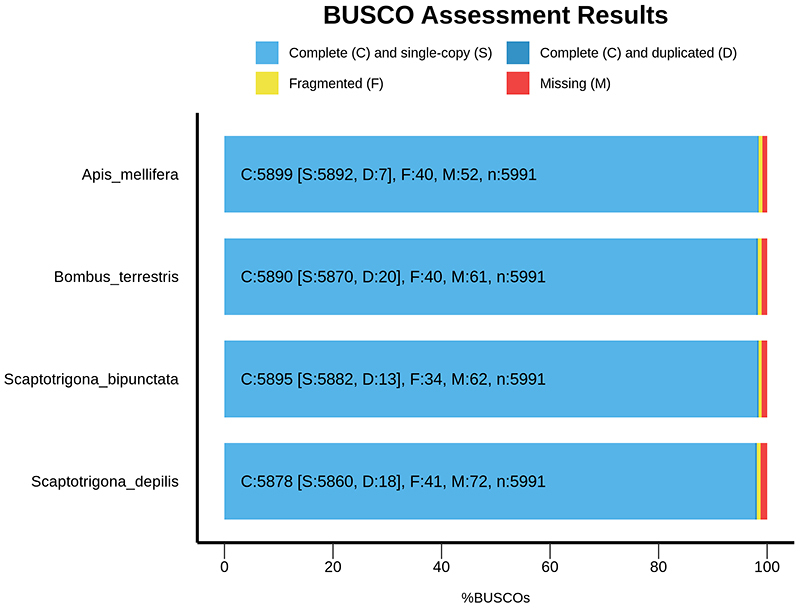
BUSCO analysis results for *Scaptotrigona bipunctata* and *S. depilis* in comparison with *Apis mellifera* and *Bombus terrestris*. The *Scaptotrigona* datasets are nearly complete and highly contiguous. The *S. bipunctata* assembly exhibits 98,4% completeness, *S. depilis* 98,1%, *Apis mellifera* 98,4%, and *Bombus terrestris* 98,3%, all of them with negligible duplication levels (<0.4%).

To evaluate genome completeness, we performed a canonical BUSCO analysis on the *Scaptotrigona* datasets, comparing them to the genomes of *Apis mellifera* and *Bombus terrestris*. Based on the BUSCO results (Figure 2), the genome assemblies for both *Scaptotrigona* species show high completeness, with 98.4% for *S. bipunctata* and 98.1% for *S. depilis*. Additionally, both assemblies exhibit a high level of contiguity, with N50 ranging from 11.3 Mb to 12.9 Mb. The genome sizes of the two stingless bee species (301 Mb and 292 Mb) fall between those of *A. mellifera* (228 Mb) and *B. terrestris* (393 Mb), despite the fact that most genes are single-copy and the number of chromosomes is quite similar, with 16 in *A. mellifera*, 17 in *S. bipunctata and S. depilis*, and 18 in *B. terrestris*.

### Genome prediction and functional annotation

Before gene prediction, both genomes were masked for repetitive regions. More than 20% of each genome was masked for both species, with the majority (15%) being unclassified, 3.35% consisting of retroelements, and 2.5% of DNA transposons. The gene prediction process generated 17,466 protein coding sequences (CDS) for *S. bipunctata* and 16,166 for *S. depilis* (Table 1). During functional annotation, 9,030 CDS sequences matched proteins in the Swissprot reference database.

Several thousand sequences however did not have hits in databases like SwissProt (blastx returned no results). Most of these sequences were relatively short (less than 150 bp) and may represent novel genes, bioinformatics artifacts, partial gene sequences, pseudogenes, or even transposons and other selfish elements that were not removed during the masking process. This matter will require further study and analysis. Moreover, a small percentage of the genes in the genome were not correctly predicted, even with RNA-seq evidence, as indicated by the BUSCO analysis of the gene prediction results (94.2% completeness for gene prediction results *vs.* 98.4% completeness for the genome assembly prior to gene prediction in the case of *S. bipunctata*). Nonetheless despite these challenges, the integration of RNA-seq data from different tissues proved essential for capturing tissue-specific and alternatively spliced transcripts, thereby significantly enhancing the overall annotation quality.

### Synteny analysis

**Figure 3 -:**
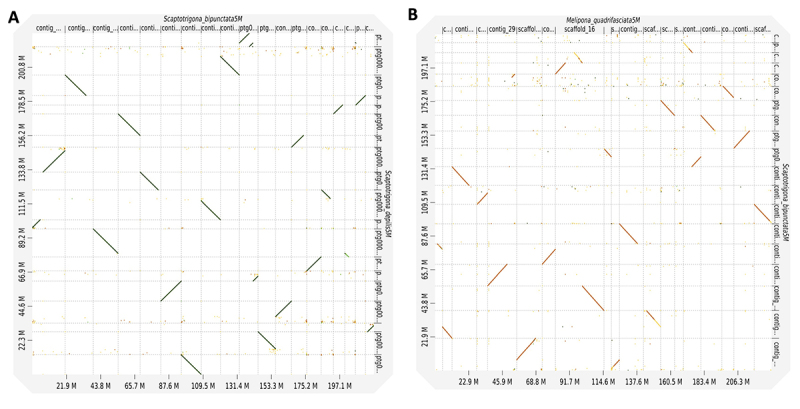
Synteny analysis results. A) Synteny analysis between the two species *Scaptotrigona bipunctata and S. depilis,* which exhibit a very strong synteny with each other. B) Synteny analysis between *Scaptotrigona bipunctata* and *Melipona quadrifasciata*, which also exhibit strong synteny to each other. Different colors reveal the level of similarity, with a darker color representing higher similarity.

The whole genome alignments confirmed the expected strong synteny between the two *Scaptotrigona* species (Figure 3A). Interestingly, we also found a considerable level of synteny between *S. bipunctata* and *Melipona quadrifasciata* (Figure 3B), which is worthy of note, considering that the two stingless bee genera are not closely related (Rasmussen and Cameron, 2010; Engel et al., 2023; Lepeco et al., 2024) and because they differ considerably in their karyotypes. Whereas the haploid *Scaptotrigona* genome is physically divided into 17 chromosomes, which is a typical number for most stingless bees (Cunha et al. 2021), the genus *Melipona* is an exception in this clade, with most species exhibiting a haploid number of only nine chromosomes. Hence, the remarkable synteny result for the comparison between *S. bipunctata* and *M. quadrifasciata* indicates that there seems to be no apparent evidence for large genomic rearrangements along the evolutionary history of the Neotropical Meliponini, besides fusions or breaks in the chromosomes. Yet, genomic information from additional species is definitely required for a better understanding of the genomic architecture, especially synteny, in relation to the karyotypic evolution in the highly diverse stingless bees.

### Phylogenomics of corbiculate bees

**Figure 4 -:**
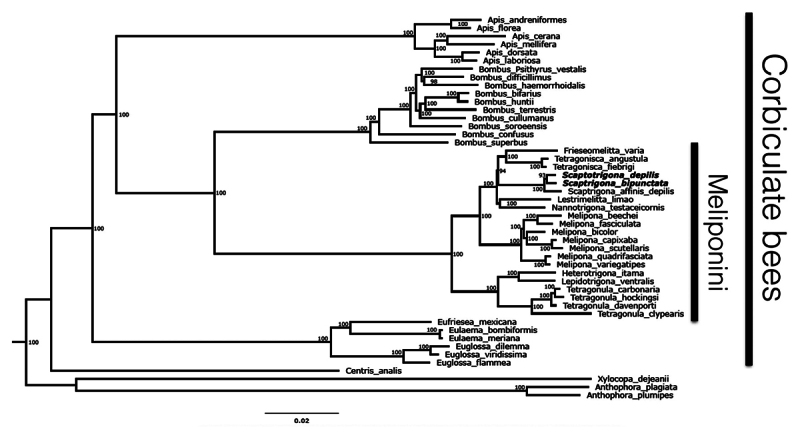
Phylogenomic representation of the clade of corbiculate bees. The four tribes (Euglossini, Apini, Bombini and Meliponini) are recovered with 100% statistical support, confirming the predicted monophyly of the clades. The members of the Meliponini fall into two clusters, one composed of Australasian species, and another one with the Neotropical representatives. Afrotropical genera are not represented due to the lack of genomic data. The phylogeny was produced from a supermatrix consisting of 1,234 concatenated genes, totaling 2,394,607 positions for 47 bee species of the clade of corbiculate bees and four bee species as outgroup. The numbers on the branches are bootstrap values representing the statistical support for each node of the tree.

New genomes provide a rich source of information for evolutionary studies. We leveraged this information to construct a statistically robust phylogenomic tree (Figure 4). This phylogenomic tree was produced from a supermatrix of 1,234 concatenated protein coding sequences (CDS), totaling 2,394,607 positions from 47 bee species within the monophyletic clade of corbiculate bees, with other members of Apidae serving as an outgroup (Texts S1 and S2). These sequences were selected because they are single copy genes, shared by all species selected for the phylogenomics analysis, and are also part of the BUSCO set of hymenopteran sequences. In the resulting tree, *Centris analis* is positioned as sister to the clade of corbiculate bees, and *Xylocopa* and *Anthophora* are the outer-most groups.

Within the corbiculate bees, the solitary to facultatively eusocial orchid bees (Euglossini) - represented in our genomic tree by *Eufriesesa mexicana*, two species of *Eulaema* and three *Euglossa* species - are the first to branch, followed by the branch consisting of the six *Apis* species for which sequenced genomes are available. The Bombini, in our tree represented by 10 species with genomic or transcriptomic data, came out with very high bootstrap support (100) as the sister group to Meliponini. Within the Meliponini, the *Scaptotrigona* species form a branch with *Frieseomelitta, Tetragonisca, Nannotrigona* and *Lestrimelitta* (Figure 4). This branch is clearly separated from the species representing the *Melipona* clade, and together, these 15 species represent the Neotropical stingless bees. Worthy of note is the presence in the tree of three different entries for *Scaptotrigona*, two of which classified as *“S. depilis”* or “*S. affinis depilis”*. This result suggests that there is some discrepancy around this scientific name, a problem that requires a more in-depth study, and especially taxonomic work.

The Neotropical Meliponini are clearly set apart from the Indo-Australasian stingless bees, represented in our tree by *Heterotrigona itama*, *Lepidotrigona ventralis*, and four *Tetragonula* species. Unfortunately, Afrotropical stingless bee genera are not represented because genomic or even transcriptomic information, to our knowledge, is still lacking for members of this lineage.

## Discussion

Even though the Meliponini form a highly diverse group of bees with pantropical distribution, only a handful of species had their nuclear genomes sequenced and published so far. Five of these are Neotropical species (Kapheim et al., 2015; de Paula Freitas et al. 2020; Koludarov et al., 2023; Araujo *et al.* 2024, Ferrari et al., 2024), and one is an Australasian species (Koludarov *et al.,* 2023). Another four Indo-Australasian species (genera *Tetragonula*, *Lepidotrigona* and *Heterotrigona*) have unpublished sequences deposited in GenBank. In this study, we now contribute to improve this picture with the first two genomes for the Neotropical genus *Scaptotrigona*. 

With 20 recognized species, *Scaptotrigona* is one of the largest genera among the Neotropical stingless bees (Camargo and Pedro, 2007). *Scaptotrigona* species tend to have large colonies, composed of several thousand workers, which aggressively defend their nests. Their workers are capable of laying eggs in the presence of the queen, thus, genetically contributing to the production of males in the colony (Vollet-Neto et al., 2018). 

*Scaptotrigona bipunctata* Latreille, 1836, popularly known as “tubuna”, is taxonomically well characterized, presenting two white spots on the abdominal tergites (Figure 1B ). In contrast, the species *Scaptotrigona depilis* Moure, 1942 is frequently found referred to in the literature as *S. postica* or *S.* aff. *depilis*. This is due to the fact that the holotype of *S. postica* has been lost, and the species itself, originally listed as *Melipona postica* by Latreille in 1806, is a *nomen nudum*. Furthermore, Camargo and Pedro (2007) mention that the name *S. postica* has been attributed to apparently different species. Recently, in a search for the respective *S. postica* type material, Melo (2023) discovered that the name *Scaptotrigona xanthotricha* Moure 1950 is actually a junior synonym to *S. postica*, and a conclusion (E.A.B de Almeida, personal communication) from an ongoing taxonomic revision of the genus *Scaptotrigona* is that *S. depilis* is a polymorphic species, with some specimens presenting yellow hair tufts on the abdomen, while in others the abdomen is glabrous. Hence, the voucher material of the species sequenced in this study, while coming from a colony that we originally considered to be *S. postica* or *S.* aff. *depilis*, was subsequently identified by E.A.B de Almeida as representing the species *Scaptotrigona depilis* Moure 1942. This issue arises explicitly in our phylogenomics analysis, as two clearly distinct entries are both labeled *S. depilis*, and are not even being sister taxa. One of these, labeled ‘S. depilis’, appears as the sister species to *S. bipunctata*, while the other, ‘*S. depilis*’, is recovered as an outgroup to both *Scaptotrigona* species. This highlights the need for further taxonomic studies within this genus, to clarify species boundaries and improve the current classification.

Comparing the genome assembly data of the four species of bees listed in Table 1, there is a notable variation in genome sizes. The reason behind is not clear, as there is no evidence that any bee species has undergone polyploidization, or that bees tolerate aneuploidy. Additionally, there is no evidence for supernumerary B chromosomes in the sampled species, although the phenomenon is described for other Meliponini species (Cunha et al. 2021). One possible reason is variation in the number of transposable elements, or expansions or reductions in repetitive regions of the genome. In contrast, functional gene duplications seem to be rare, as duplication levels pointed out by the BUSCO analysis are less than 1% for all genes searched by the pipeline. Adding to the complexity of this issue, there are currently three different genome assemblies available for *Bombus terrestris*, with sizes ranging from 248 Mb to 393 Mb. Similar discrepancies in genome size are, however, frequently observed, and can eventually be solved via pangenomics approaches (Golicz et al., 2020; Eynard et al., 2024). The datasets for the genera *Bombus* and *Scaptotrigona* basically do not differ with respect to CG content, but together, they present a higher CG content in comparison to the genus *Apis*. This may have implications for epigenetic factors and chromatin accessibility, as gene body methylation in bees takes place in the CpG dinucleotide context (Wang et al., 2006). 

With respect to our phylogenomic analysis (Figure 4), the resulting tree is in conformity with the recent phylogenomic analysis by Lepeco et al. (2024), generated by sequencing ultraconserved elements (UCEs) from DNA extracted from 153 Meliponini species, including Neotropical, Indo-Australasian, and Afrotropical genera, as well as species of the genus *Bombus*, *Apis*, and four Euglossini (genera *Eufriesea*, *Eulaema*, *Euglossa*, and *Aglae*). Both in our tree and in this UCEs-based tree, *Centris analis* came out as outgroup to the corbiculate bees. In our phylogenomic analysis, the two newly sequenced *Scaptotrigona* species clustered with *Tetragonisca, Frieseomelitta, Nannotrigona and Lestrimellitta,* and are clearly separated from the *Melipona* clade. In essence, this is a simplified version of the results obtained by Lepeco *et al.* (2024), which included more species and, within the Neotropical Meliponini, grouped *Scaptotrigona* with *Oxytrigona*, *Trigona*, *Geotrigona*, *Cephalotrigona* and some smaller genera, forming a ‘Trigona clade’. Our analysis also provides strong support for the monophyly of the corbiculate bees, as well as the monophyly of all groups within the clade. All these branches have now 100% statistical support due to the large amount of concatenated sequence data that comprised the supermatrix that we generated for building the tree. 

Our phylogenomic tree is in full agreement with the proposals of Rasmussen and Cameron (2010) and Lepeco et al. (2024), all exhibiting a monophyletic clade with the same relationships among its groups (Euglossini(Apini(Bombini+Meliponini))). The evolution of eusociality originated in the ancestor of the clade ABM (Apini+Bombini+Meliponini), while the Euglossini diverged earlier and remained solitary or facultatively eusocial. The separation of Apini from Bombini+Meliponini, furthermore, is consistent with the view that advanced eusociality evolved independently in the honey bees and the stingless bees. Nonetheless, in a multidimensional analysis (Holland and Bloch, 2020), the Bombini, which are generally classified as primitively eusocial (Michener, 2007), came out very close to the Meliponini in many aspects of their colony parameters, hence calling into question the independent evolution of advanced eusociality in the Apini and Meliponini. An alternative scenario is that the whole clade ABM is ancestrally eusocial, but that the Bombini simplified their social organization secondarily as an adaptation to survive the harsh winters of colder regions, which they indeed colonized successfully. Solving this and other questions will definitely require a significant increase in the number of sequenced and well annotated stingless bee genomes.

Supplementary material

The following online material is available for this article:

Figure S1 - FindGSE estimation of the genome size of *Scaptotrigona bipunctata*.

Figure S2 - FindGSE estimation of the genome size of *Scaptotrigona depilis*.

Figure S3 - OGDraw representation of the assembly and annotation of the mitogenome of *Scaptotrigona bipunctata*.

Figure S4 - OGDraw representation of the assembly and annotation of the mitogenome of *Scaptotrigona depilis*.

Table S1 - Genome assemblies and transcriptome data used for the matrix construction of the phylogenomics analysis.

Text S1 - Supermatrix with concatenated alignments of 1,234 CDS, totaling 2,394,607 positions. The supermatrix consisting of 1234 concatenated CDS used for the construction of the phylogenetic tree is available at Zenodo: https://zenodo.org/records/15333463.

Text S2 - Text representation of the consensus tree produced from the supermatrix.

## Data Availability

 The two genomes are deposited in GenBank under the accession numbers SAMN44369367 (*S. bipunctata*), SAMN44369368 (*S. depilis*). The mitochondrial genome of *S. bipunctata* is deposited in GenBank under the accession number PV624662 and that of *S. depilis* as PV660477.
