# Supplementary material for: The genomes sequenced for the neotropical stingless bees Scaptotrigona bipunctata and S. depilis strengthen the phylogenomics support for the taxonomy of social bees
Source: Genet Mol Biol. 2025 Nov 28;48(4):e20240255. doi: 10.1590/1678-4685-GMB-2024-0255 (PMC12703582; doi:10.1590/1678-4685-GMB-2024-0255)
Supplement: Figure S4 - [file 1415-4757-GMB-48-04-e20240255-s4.pdf]

Genetics and Molecular Biology

https://doi.org/10.1590/1678-4685-GMB-2024-0255

Supplementary Material to "The genomes sequenced for the neotropical stingless bees *Scaptotrigona bipunctata* and *S. depilis* strengthen the phylogenomics support for the taxonomy of social bees"

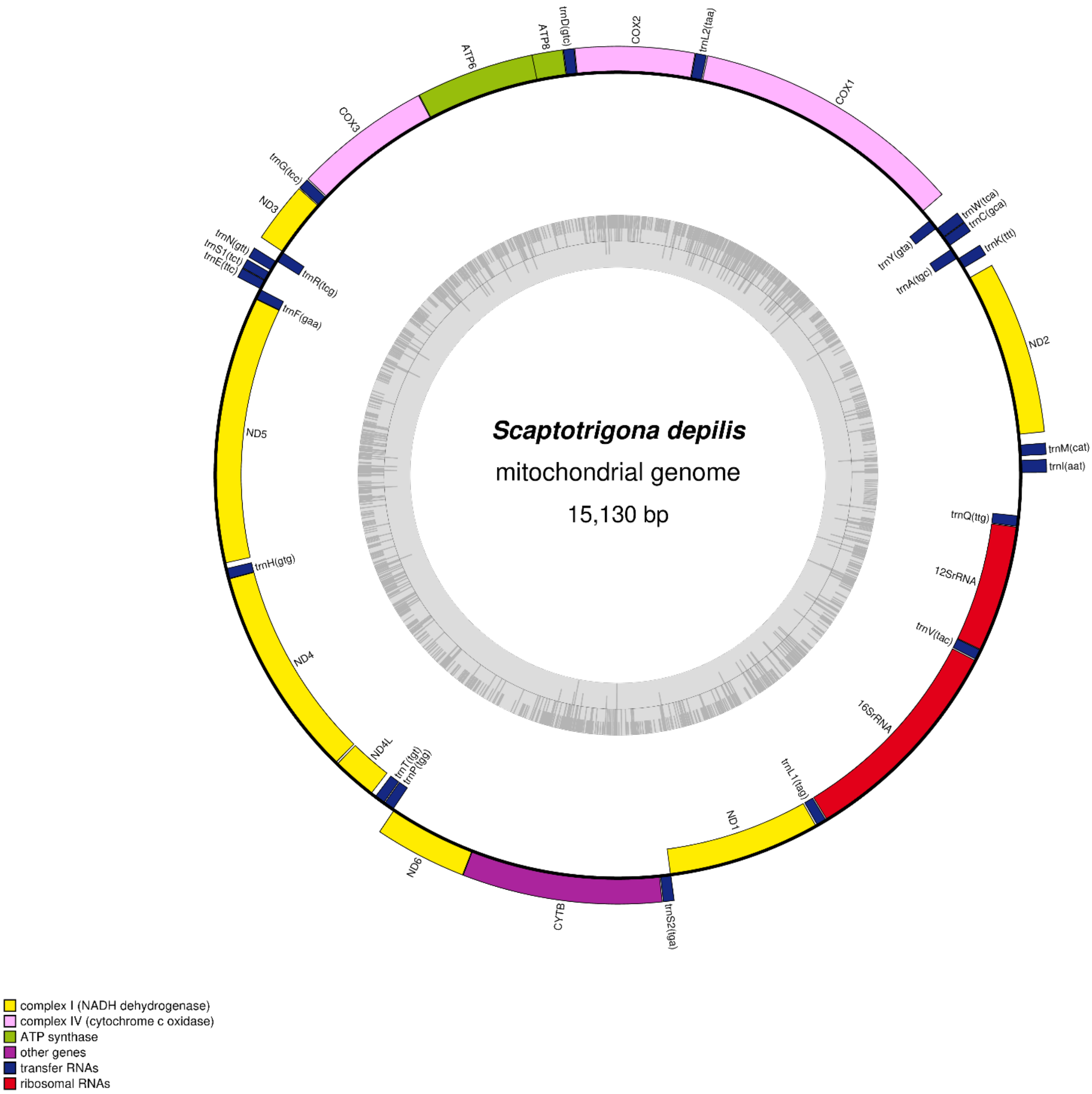

Figure S4 - OGDraw representation of the assembly and annotation of the mitogenome of *Scaptotrigona depilis*.
