# Supplementary material for: The genomes sequenced for the neotropical stingless bees Scaptotrigona bipunctata and S. depilis strengthen the phylogenomics support for the taxonomy of social bees
Source: Genet Mol Biol. 2025 Nov 28;48(4):e20240255. doi: 10.1590/1678-4685-GMB-2024-0255 (PMC12703582; doi:10.1590/1678-4685-GMB-2024-0255)
Supplement: Table S1 - [file 1415-4757-GMB-48-04-e20240255-s5.pdf]

Genetics and Molecular Biology
https://doi.org/10.1590/1678-4685-GMB-2024-0255

Supplementary Material to "The genomes sequenced for the neotropical stingless bees *Scaptotrigona bipunctata* and *S. depilis* strengthen the phylogenomics support for the taxonomy of social bees"

Table S1 - Genome assemblies and transcriptome data used for the matrix construction of the phylogenomics analysis.

Species	Assembly	GenBank /Biosample ID	RefSeq
Apis dorsata	Apis dorsata 1.3	GCA_000469605.1	GCF_000469605.1
Apis laboriosa	ASM1406632v1	GCA_014066325.1	GCF_014066325.1
Apis mellifera	Amel_HAV3.1	GCA_003254395.2	GCF_003254395.2
Apis cerana	AcerK_1.0	GCA_029169275.1	GCF_029169275.1
Apis florea	Aflo_1.1	GCA_000184785.2	GCF_000184785.3
Apis andreniformes	ASM4859352v1	GCA_048593525.1	
Bombus (Psithyrus) vestalis	iyBomVest1.1	GCA_963556215.1	
Bombus difficillimus	ASM1473752v1	GCA_014737525.1	
Bombus haemorrhoidalis	ASM1482597v1	GCA_014825975.1	
Bombus cullumanus	ASM1473753v1	GCA_014737535.1	
Bombus huntii	iyBomHunt1.1	GCA_024542735.1	GCF_024542735.1
Bombus bifarius	Bbif_JDL3187	GCA_011952205.1	GCF_011952205.1
Bombus terrestris	iyBomTerr1.2	GCA_910591885.2	GCF_910591885.1
Bombus soroeensis	ASM1473736v1	GCA_014737365.1	
Bombus confusus	ASM1473747v1	GCA_014737475.1	
Bombus superbus	ASM1473738v1	GCA_014737385.1	
Frieseomelitta varia	Fvar_1.2	GCA_011392965.1	GCF_011392965.1
Tetragonisca angustula	jatai_v2	GCA_036937485.2	
Tetragonisca fiebrigi	transcriptome	PRJNA1021589	
Scaptotrigona bipunctata	this study	to be released	
Scaptotrigona depilis	this study	to be released	
Scaptotrigona affinis depilis	transcriptomes	PRJNA1113344	
Lestrimelitta limao	Transcriptomes	PRJNA1113344	
Nannotrigona tetstaceicornis	Transcriptomes	PRJNA1113344	
Melipona beechei	MelBeec_1.0	GCA_032399605.1	
Melipona fasciculata	transcriptome	PRJNA922095	
Melipona bicolor	USP_Mbic_1.2	GCA_030673865.1	
Melipona quadrifasciata	ASM127656v1	GCA_001276565.1	
Melipona variegatis	transcriptome	SAMN44369367	
Melipona scutellaris	transcriptome	PRJNA1026046	
Melipona capixaba		(SRA) ERR14763069	
Heterorigona itama	IQG0107_L5	GCA_903986555.1	
Lepidotrigona ventralis	ASM280687v1	GCA_002806875.1	
Tetragonula carbonaria	TetCarb_2.0	GCA_032399595-1	

Tetragonula hockingsi	Tetragonula_hockingsi_v1.1	GCA_010645185.1	
Tetragonula davenporti	Tetragonula_davenporti_v1.1	GCA_010645165.1	
Tetragonula clypearis	Tetragonula_clypearis_v1.1	GCA_010645135.1	
Eufriesea mexicana	ASM148370v2	GCA_001483705.2	GCF_001483705.2
Eulaema meriana	transcriptome	PRJNA859127	
Eulaema bombiformis	transcriptome	PRJNA387619	
Euglossa viridissima	raw sequence reads	PRJNA529235	
Euglossa dilemma	Edil_v1.0	GCA_002201625.1	
Euglossa flammea	transcriptome	PRJNA387619	
Centris analis	transcriptome and UCEs	PRJNA529235	
Xylocopa dejeanii	ASM4900475v1	GCA_049004755.1	
Anthophora plagiata	IyAntPlag1	GCA_963995485.1	
Anthophora plumipes	iyAntPlum1.1	GCA_951804975.1	
