# Supplementary material for: The genomes sequenced for the neotropical stingless bees Scaptotrigona bipunctata and S. depilis strengthen the phylogenomics support for the taxonomy of social bees
Source: Genet Mol Biol. 2025 Nov 28;48(4):e20240255. doi: 10.1590/1678-4685-GMB-2024-0255 (PMC12703582; doi:10.1590/1678-4685-GMB-2024-0255)
Supplement: Text S2 - [file 1415-4757-GMB-48-04-e20240255-s7.pdf]

*Genetics and Molecular Biology*

https://doi.org/10.1590/1678-4685-GMB-2024-0255

# Supplementary Material to “The genomes sequenced for the neotropical stingless bees *Scaptotrigona bipunctata* and *S. depilis* strengthen the phylogenomics support for the taxonomy of social bees”

**Text S2 -** Text representation of the consensus tree produced from the supermatrix.

(Anthophora_plagiata_13026:0.0169160735,Anthophora_plumipes_41017:0.0146382314,((((((Apis_andreniformes_7646:0.0081993517,Apis_florea_11378:0.0052089551)100:0.0099856705,((Apis_cerana_469:0.0159151493,Apis_mellifera_11022:0.0127312591)100:0.0035283264,(Apis_dorsata_10895:0.0047144261,Apis_laboriosa_6355:0.0039305925)100:0.0075194222)100:0.0054713135)100:0.0816679422,((((((Bombus_Psithyrus_vestalis_6801:0.0137676984,(Bombus_difficillimus_844:0.0107726982,Bombus_haemorrhoidalis_7209:0.0147970226)98:0.0006032964)100:0.0015027840,(((Bombus_bifarius_2147:0.0027761612,Bombus_huntii_18056:0.0026323472)100:0.0089454780,Bombus_terrestris_9426:0.0138631272)100:0.0014987453,Bombus_cullumanus_13814:0.0124101221)100:0.0010363326)100:0.0015578145,Bombus_soroeensis_13976:0.0139677352)100:0.0086328738,Bombus_confusus_5113:0.0208859420)100:0.0024325031,Bombus_superbus_8035:0.0212773708)100:0.0425876902,(((((Frieseomelitta_varia_6647:0.0139281906,(Tetragonisca_angustula_27:0.0017448777,Tetragonisca_fiebrigi_214:0.0012666572)100:0.0096813384)100:0.0023015950,((Scaptotrigona_postica2499:0.0025221916,Scaptrigona_bipunctata_5812:0.0026290396)93:0.0006172879,Scaptrigona_depilis_160:0.0046754791)100:0.0127079051)94:0.0003881938,(Lestrimelitta_limao_364:0.0138924994,Nannotrigona_testaceicornis_119:0.0123641896)100:0.0009681789)100:0.0045326417,(((Melipona_beechei_.codingseq7589:0.0064933070,Meliponafasciculata_.codingseq2491:0.0041717024)100:0.0037692619,(Melipona_bicolor_7184:0.0050101271,(Melipona_capixaba_12567:0.0011586906,Melipona_scutellaris_134:0.0029819265)100:0.0069009864)100:0.0008272299)100:0.0006158014,(Melipona_quadrifasciataLocal_10928:0.0013640341,Melipona_variegatipesConsensus_19495:0.0012275900)100:0.0066740496)100:0.0113397306)100:0.0077447748,((Heterotrigona_itama7906:0.0100059259,Lepidotrigona_ventralis_22045:0.0098463562)100:0.0056965511,(((Tetragonula_carbonaria_1726:0.0014646569,Tetragonula_hockingsi_4413:0.0025114000)100:0.0009803207,Tetragonula_davenporti_2794:0.0014184455)100:0.0053542978,Tetragonula_clypearis_7508:0.0162486893)100:0.0092263776)100:0.0126809761)100:0.0648754566)100:0.0268936372)100:0.0065145041,((Eufriesea_mexicana_26085:0.0220921837,(Eulaema_

bombiformis_609:0.0006962313,Eulaema_meriana_465:0.0006565137)100:0.0245370785)100:0.0052536629,((Euglossa_dilemma_4056:0.0051366950,Euglossa_viridissima_38:0.0021930105)100:0.0074110079,Euglossa_flammea_3891:0.0073032114)100:0.0198002082)100:0.0652766386)100:0.0113636867,Centris_analis_268:0.0788533847)100:0.0205941909,Xylocopa_dejeanii_6050:0.1408475522)100:0.1232208576);
